# Poisons and Poisoners

**Published:** 1891-04-04

**Authors:** 


### Poisons and Poisoners.

### I.—ARSENIC: MADELINE SMITH.

Not long ago, when a famous case of arsenic poisoning was before the public, the case of Madeline Smith, forgotten for thirty years, was recalled, and some people asked why if Miss Smith was acquitted another woman, against whom the evidence was no stronger, should be condemned. But apart from the general truism that two blacks do not make a white, it should be remembered that Madeline Smith was not really acquitted, but escaped under the Scotch verdict of "not proven," by which a prisoner may be said to leave the court with an indelible stain on his character; and that the satisfaction with which this "canny" decision was received by the public was due more to sympathy with the situation of a young girl, driven to desperation by fear of the possible action of a lover whom she had ceased to love, than to any real conviction of her innocence.

Madeline Smith was just twenty-one years of age when she was tried for the murder of her lover, a young Frenchman, named Pierre Emile L'Angelier. She was the daughter of an architect, and lived with her parents in Blythswood Square, one of the best districts in Glasgow. Her whole ancestry, however, was not of the class of *haute bourgeoisie* indicated by these facts. Her maternal grandmother was of gipsy blood, and the girl's whole conduct indicates that combination of lawless passion with lawless hate which we associate with the Romany race. A memory of "Carmen" runs through the story—a lover clung to at any price for a time, only to be thrown aside at last for another. But the *dénouement* is different from that of Mérimée's romance and Bizet's opera.

Madeline Smith had just returned, a girl of eighteen, from a London boarding-school, when in 1854 she met L'Angelier. She was pretty, lively, romantic, and inexperienced; he was good-looking, well-mannered, and plausible—a boaster, indeed, and lacking in principle, but none the less the type of man to catch a young girl's fancy. A love affair, harmless enough to begin with, sprang up between them, but Madeline's father refused his consent to a marriage, and after that intercourse and correspondence were carried on clandestinely. From Mr. Smith's point of view, a merchant's clerk was no fitting match for his daughter, and the fact of L'Angelier's nationality would not tend to soften his heart. The father's opinion of his daughter's suitor was a just one, as circumstances fully showed, and the best verdict on L'Angelier's character is the fact that the French Consul in Glasgow, M. de Meau, though he had known him in his—De Meau's—bachelor days, dropped his acquaintance when he married. Still, selfish and unscrupulous as he was, L'Angelier's love for Miss Smith seems to have been of its kind, sincere. For more than two years their meetings went on, and an elopement was planned, but never carried out. Suddenly Madeline's affection waned. To all appearance she was the same; her letters were still full of phrases, that to some men would have been nauseating in their effusiveness, but from outside sources he heard of a contemplated marriage with another man, and when questioned on the subject Madeline could not deny the truth of the report.

Mr. Minnoch, of the well-known firm of W. Houldsworth and Co., was a very different aspirant for Madeline Smith's hand from the French adventurer, L'Angelier. To his suit a father's consent and a mother's approval were easily obtained, and when, on January 28th, 1857, Madeline accepted him as her future husband, everything seemed smooth and prosperous. But L'Angelier was to be reckoned with, and he held in his possession two hundred letters of hers, many of which were of such a character as to deter any self-respecting man from making the writer of them his wife. To obtain these letters now became her most earnest desire. But L'Angelier would not part with them. By means of them he held her in his power, and he gave her the choice between becoming his wife and having these letters laid before her father. At any price, he was determined to prevent her marrying Minnoch. How much she feared him, how bitterly in earnest she was in her desire to be free of his toils is shown by a letter she wrote to him on February 11th, 1857. "As you hope for mercy at the judgment day," she prays him, "do not inform on me; do not make me a public shame. There is no one I love. My love was all given to you. My heart is empty, cold. I am unloved. I am despised. I told you I had ceased to love. It is true." In a postscript she makes an appointment for him to come to her window, which was on the ground floor, at twelve o'clock the following night. Apparently she hoped that a personal appeal would soften his heart. Whether or not this interview took place is uncertain; but if it did, neither that nor any subsequent meeting availed to move L'Angelier's resolution.

On March 18th Madeline and her parents were to dine a Mr. Minnoch's house. That morning she bought six pennyworth of arsenic. She told the chemist from whom she bought it that it was meant to destroy the rats that infested her father's country house at Rowaleyn, in one of the villages that nestle on the picturesque coast of the Clyde. The explanation was false; but this in itself is no conclusive proof of evil intent. Her subsequent statement that she meant to use the arsenic as a cosmetic to improve her complexion for that evening's party was plausible enough. A girl will rarely confess to the use of such things, however largely she may indulge in it. It is true that arsenious acid, the common arsenic of the shops, which was the form in which she obtained it, is valueless as a cosmetic, but of this again she might easily be ignorant. According to her own statement she mixed the arsenic with the water she washed in, and bathed her face and arms with it. According to the opinion of Dr. Frederick Penny, Professor of Chemistry in the University of Glasgow at the time, such a use of arsenic was likely to have produced an eruption on the skin; but, on the other hand, Dr. (now Sir) Douglas Maclagan, of Edinburgh University, gave it as his opinion that it would be perfectly harmless. Nothing was gained from the evidence of the medical experts on this point.

(*To be concluded.*)

